# Clinical aspects of acute pneumonia in Bihor county

**DOI:** 10.1186/1471-2334-14-S7-P18

**Published:** 2014-10-15

**Authors:** Andrei Csep

**Affiliations:** 1 Faculty of Medicine and Pharmacy, University of Oradea, Romania

## Background

In developing countries, acute pneumonia is a very important public health problem.

## Methods

Our goal was to study all the 420 patients with acute pneumonia who were admitted to the Infectious Clinic in Oradea between 01.01.2013-31.12.2013. The diagnosis was performed based on clinical, biological and radiological criteria.

## Results

Most of the patients came from rural area (60%). 55% of them were female and 45% were male. We had 3 groups of acute pneumonia: lobar pneumonia (39%), interstitial pneumonia (47%) and bronchopneumonia (14%). More than half of the patients had bacterial pneumonia (70%). From these, 68% had unknown etiology. The most affected ranges were the age group 51-55 and 61-65. Most cases of acute pneumonia were diagnosed in autumn. Associated pathology was observed in old patients (47%)

## Conclusion

The most exposed category of age was represented by adults between 51 and 55 years and 61 and 65 years old.

